# Robotic-Assisted Thoracic Surgery Approach to Thoracic Endometriosis Syndrome with Unilateral Diaphragmatic Palsy

**DOI:** 10.1155/2023/5493232

**Published:** 2023-08-22

**Authors:** Abiah Jacob, Adeyemi Coker, Steven Aleksandar Stamenkovic

**Affiliations:** ^1^Barts Thorax Centre, St. Bartholomew's Hospital, Barts Health NHS Trust, West Smithfield, London EC1A 7BE, UK; ^2^Queens University Hospital Endometriosis Centre, Barking Havering and Redbridge University Hospitals NHS Trust, Romford RM7 0AD, UK; ^3^Advanced Laparoscopic Gynaecology, Barts Health NHS Trust, West Smithfield, London, UK

## Abstract

Endometriosis is characterized by endometrial-like glands and stroma outside the uterine cavity, affecting women of reproductive age. Thoracic endometriosis syndrome (TES) is an entity producing a range of clinical and radiological manifestations, including catamenial pneumothorax, haemothorax, haemoptysis, and pulmonary nodules within the thoracic cavity or on the diaphragm. TES symptoms are nonspecific, warranting a high degree of clinical suspicion. Management includes hormone replacement therapy, surgical management, or a combination of both. We present a case of a 37-year-old woman who presented with TES and unilateral diaphragmatic palsy, managed with robotic-assisted thoracoscopic surgery and hormone replacement.

## 1. Introduction

Thoracic endometriosis syndrome (TES) is a rare form of extrapelvic endometriosis with significant clinical implications. It is rarely seen outside the setting of an endometriosis centre where the multidisciplinary approach and high index of suspicion facilitate its timely diagnosis and treatment.

The diagnosis is often delayed or missed, resulting in recurrent hospitalizations and other complications. A woman in her reproductive years with chest pain, dyspnoea, or cough around the time of her menstrual cycle should raise a possible diagnosis of TES. Physical examination may reveal absent or diminished breath sounds on the affected side, suggesting pleural effusion and/or pneumothorax. Chest radiography may show pleural effusions, pneumothorax, or pulmonary nodules. Both computerized tomography (CT) and magnetic resonance imaging (MRI) aid in the diagnosis of TES. CT may show hypo-attenuating areas revealing diaphragmatic implants and help in identifying single or multiple pulmonary nodules [1].

The true prevalence and age incidence of thoracic endometriosis (TE) remains unknown and while it is considered rare, this may represent a largely underdiagnosed disease [2]. Nezhat et al. reported that up to 80% of women with TE have accompanying abdominopelvic endometriosis [3].

The most common site of endometriosis outside of the abdominopelvic cavity is the thorax. Endometriosis within the lung parenchyma, pleural surfaces, or on the diaphragm produces a range of clinicoradiological manifestations, such as catamenial pneumothorax, haemothorax, haemoptysis, and pulmonary nodules, collectively known as TES. Joseph and Sahn's retrospective analysis reported: catamenial pneumothorax as the most frequent (73% of published cases of TES); catamenial haemothorax (14%); catamenial haemoptysis (7%); and endometriotic lung nodules (6%). Isolated catamenial pneumomediastinum and chest pain were found in only one case each [4]. Recent literature includes other entities of TES: endometriosis-related diaphragmatic hernia, catamenial chest pain, and endometriosis-related pleural effusion [5].

The prevalence of this condition has led the British Society for Gynaecological Endoscopy (BSGE) and Royal College of Obstetricians and Gynaecologists to release an official statement to both increase awareness of the condition and attempt to standardise care [2].

The diagnostic and curative pathways of TES are based on the disease manifestation and determined by both thoracic surgeons and gynaecologists. Nezhat et al. reported that a single procedure with a multidisciplinary surgical approach combining video laparoscopy and video-assisted thoracoscopic surgery (VATS) performed by gynaecologic and thoracic surgeons who are familiar with endometriosis provides an effective result [6].

A meta-analysis and review of patients with TES by Ciriaco et al. found that the majority of patients underwent VATS for surgical management [7]. A retrospective observational study conducted by Quercia et al. examined their experience using VATS and recommended this approach. The authors found it to be useful for obtaining an exploratory diagnosis of ectopic endometrial implants and diaphragmatic fenestrations, as well as obtaining appropriate surgical treatment and pathological specimens to confirm the diagnosis [8]. However, in recent years the field of robotic thoracic surgery has rapidly evolved and has gained widespread use in thoracic centres around the world.

The robotic system gives several advantages over standard VATS. This includes enhanced highly-magnified 3D high-definition (3DHD) vision and true depth perception allowing clear identification of structures and tissue planes. In addition, the multi-functional wristed robotic instruments with seven degrees of freedom permit a greater range of motion and allow complex yet safe surgical manoeuvres even in narrow spaces as stated by Ricciardi et al. [9]. Farivar et al. stated that the tremor reduction filter (6-Hz motion filter) in robotic-assisted thoracoscopic surgery (RATS) counteracted physiologic hand tremors and permitted precise and accurate movements resulting in fine surgical manoeuvers. This feature allows better dissection, reduced blood loss, and accurate suturing [10]. In addition, the use of intra-pleural carbon dioxide pushes structures away, including the lung and diaphragm permitting a larger space to work in, especially at the base of the chest. Finally, the robotic system allows for improved ergonomics and reduces surgeon and muscular fatigue since it allows the surgeon to sit comfortably as opposed to VATS where the surgeon assumes an exhausting, standing posture. These features make RATS a preferred choice over VATS where the lack of instrument articulations and technical difficulties amplify unintended surgical movements [9].

We present a case of a 37-year-old woman who presented with TES and right unilateral diaphragmatic palsy, treated with RATS and hormone replacement therapy (HRT). The purpose of this procedure was to perform an exploratory diagnosis and obtain pathological specimens for a definitive diagnosis while also providing symptomatic relief.

## 2. Case Presentation

A 37-year-old female presented with progressive dyspnoea, reduced exercise tolerance, cyclical chest pain, cyclical abdominal pain, and generalised fatigue.

At the endometriosis multidisciplinary team follow-up, she was suspected of having TE based on MRI and CT scans had an established diagnosis of pelvic endometriosis and treatment for Grade 4 pelvic endometriosis at the endometriosis centre. The American Reproductive Society of Medicine staging for endometriosis was used to stage her disease (Table 1). She was referred first to the local respiratory team with catamenial haemothorax and haemoptysis, and then to the tertiary Barts Thorax Centre.

**Figure 1:**
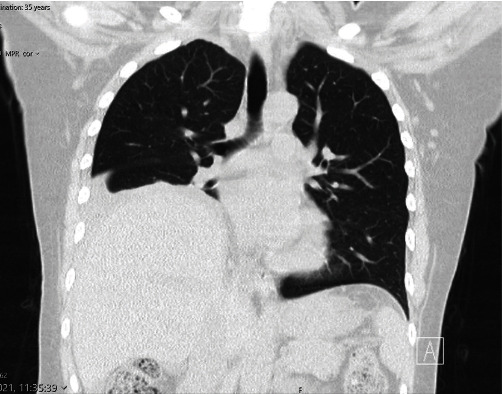
HRCT thorax right pleural effusion and raised right hemidiaphragm. HRCT: high-resolution computed tomography.

MRI pelvis showed active pelvic endometriosis and chronic endometriosis. High-resolution CT thorax showed right pleural effusion and a raised right hemidiaphragm, suggesting phrenic nerve palsy (Figure 1). MRI Cervical spine showed multilevel degenerative changes without any cord compression or significant neural foraminal stenosis, concluding early cervical spondylosis.

Ultrasound and videofluoroscopy confirmed right diaphragmatic paralysis. Spirometry showed decreased forced expiratory volume (FEV1 75.8%) and forced vital capacity (FVC 81.2%). FEV 1% FVC was 98.2%. Maximal inspiratory peak pressure was also reduced (31.8%) indicating diaphragmatic weakness.

**Figure 2:**
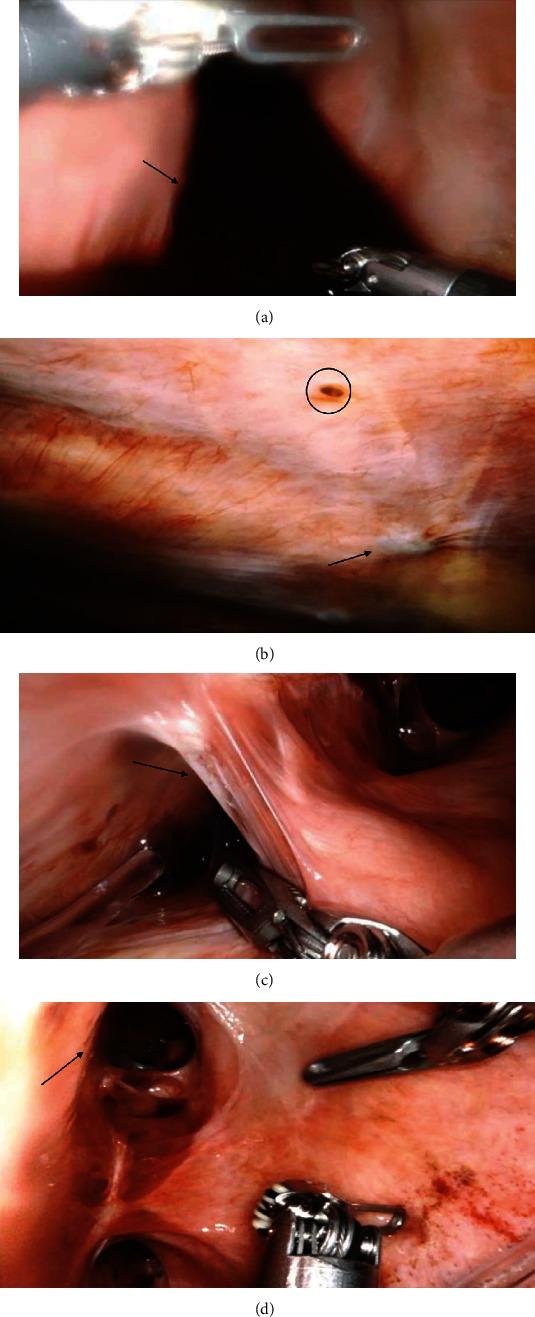
(a) RATS exploration showing haemorrhagic effusion; (b) endometrial chocolate lesion over the pleural surface (circled) and healed lesion (arrow); (c) adhesions (arrow) between the right lung and the diaphragm; (d) diaphragmatic fenestrations (arrow). RATS: robotic-assisted thoracic surgery.

**Figure 3:**
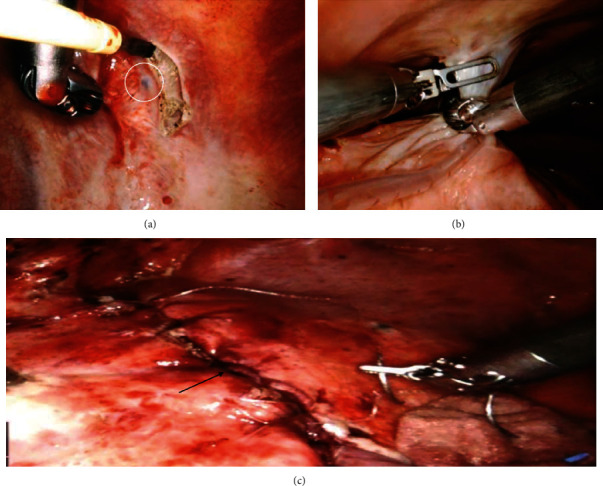
(a) Endometrial lesion biopsy; (b) adhesiolysis; and (c) diaphragm plication.

The decision was made to perform a RATS drainage of effusion, diagnostic pleural biopsy, and plication of the right hemidiaphragm. She had single lung ventilation and was positioned in a left lateral decubitus position with a significant break in the table to give a 30° angle between shoulder and hip. She underwent 3 port RATS exploration using the DaVinci Xi® robotic surgical system, which revealed a haemorrhagic effusion (Figure 2(a)) and chocolate endometrial lesions on the parietal and diaphragmatic surfaces (Figure 2(b)). There were multiple adhesions between the right lung and the diaphragm and between the diaphragm and the chest wall with diaphragmatic fenestrations (Figures 2(c) and 2(d)). The presence of dense anterior adhesions restricted our ability to safely inspect the phrenic nerve for ectopic endometrial tissue. A litre of haemorrhagic fluid was drained, and several of the pleural lesions were biopsied and sent for histology (Figure 3(a)). Adhesiolysis mobilised the lung from the diaphragm (Figure 3(b)), and the diaphragm was plicated using a barbed non-absorbable one suture. (Figure 3(c)). The surgery was uneventful and lasted for an hour and 35 minutes.

She had a quick recovery and was discharged home on day 3 postoperation. Pleural fluid cytology showed no endometrial cells. Histopathology revealed pleural fibroadipose tissue with foci of endometrial glands and stroma. Immunohistology confirmed ER+, with PAX8+ in the glands and CD10+ in the surrounding stroma, consistent with a diagnosis of TES. She had symptomatic relief with reduced dyspnoea and better exercise tolerance. Her cyclical chest pain was considerably reduced. Postoperatively, she was under follow-up in the endometriosis centre and was treated with HRT with a Gonadotropin hormone-releasing hormone (GnRH) analogue (Goserelin). Within a few months of treatment, she discontinued HRT leading to a recurrence of TES symptoms.

## 3. Discussion

Several theories exist regarding the pathophysiology of TES, however, a single theory cannot account for all clinical manifestations of TES, suggesting a multifactorial etiology [6]. A retrospective study of 25 case series noted that the mean age of diagnosis of TES is 37.7 years, with a mean time lapse of 10 years from the initial diagnosis of pelvic endometriosis, indicating that either the thoracic component takes longer to develop or that TES is under-diagnosed because of a lack of detailed history taking during the initial consultation [4]. Our patient was 37 years old with a time lapse of 7 years between the diagnosis of pelvic endometriosis and TES.

Minimally invasive surgery is considered the gold standard in diagnosing TES [11]. Due to concurrent pelvic and thoracic symptoms in these patients, it is paramount to assess and treat all areas affected. A collaborative approach of VATS and traditional laparoscopy optimally addresses pelvic, diaphragmatic, and TE in a single operation [4]. Once the lesions have been identified intra-operatively, targeted treatment may be carried out. Although VATS is considered the gold standard for the diagnosis and treatment of TES, particularly in catamenial pneumothorax, two cases of RATS have been reported in its management [12]. Recently, Bachi et al. reported a combined robotic-assisted laparoscopic and thoracic approach in the management of diaphragmatic, pleural, and pericardial endometriosis [13].

There are well-established benefits of a minimally invasive approach, whether by RATS or VATS. A systematic review and meta-analysis by Liu et al. showed that patients undergoing minimally invasive surgery had a shorter length of stay, less estimated blood loss, lower 30-day mortality, and a higher overall survival [14].

Mazzei and Abbas discussed their rationale for the adoption of the robotic approach while comparing RATS versus VATS. The advantages of RATS over VATS are the fully articulating instruments with a range of motion similar to the human wrist versus the long and rigid VATS instruments, which act as a fulcrum at the surgical incision causing mechanical constraints. VATS also has a two-dimensional image leading to challenges in depth perception in comparison with the 3DHD RATS imaging with true depth perception allowing for clear vision of anatomical structures [15].

Additionally, Veronesi stated that in redo operations, or where multiple thick inflammatory adhesions are present in the pleural spaces, robotic technology proves itself with enhanced vision and greater dexterity [16].

Stamenkovic and Melfi further elaborate on the benefits of the RATS approach: the fourth generation robot has four identical arms, allowing for the interchange of the camera. The “fourth arm” instruments provide sufficient tissue retraction, which means the other two arms can operate proximity nearby. In contrast, most VATS procedures require the use of one hand and the pushing of tissues. In terms of tactile feedback, they stated that 3DHD technology eliminates the need for tactile feedback by providing visual cues to distinguish between different densities of tissues, such as blocking, deformation, and blanching. By using these visual cues, instruments can be used with greater precision. Additionally, they found that patient safety with the RATS approach is ensured by excellent, clear communication between team members, specifically between the console surgeon and the first assistant. They also recommend that an emergency scenario rehearsal system should be in place before operating together as a team, in order to ensure the effectiveness of the response to significant airway problems and bleeding issues when they occur [17].

There is a higher cost of robotic procedures as compared with VATS. This has also been addressed by Stamenkovic and Slight who concluded that the resource implication benefits of RATS concerning the shorter length of hospital stay, fewer complications, enhanced recovery, and increased uptake of adjuvant treatments contribute towards decreased costs. Currently, there is a commercial expansion of the robot platform market, which may result in a competitive reduction of costs [18]. This has been further studied in Shanahan et al.'s comprehensive micro-cost analysis of RATS procedures [19].

To the best of our knowledge, this is the only case described in the literature where a RATS approach was undertaken in the management of catamenial haemothorax and unilateral diaphragmatic palsy. Many of the robotic technology features facilitated our operation. The 3DHD biscopic vision gave excellent view and depth perception. The articulated energy devices expedited the adhesiolysis. The procedure was totally endoscopic utilising moderate carbon dioxide insufflation, which meant the hemidiaphragm could be pushed away allowing excellent vision of a loose central tendon, and good purchase of the tissue edges. The articulated needle holder and forceps made the diaphragm plication very easy. We were unable to confirm endometrial lesions in the anterior hilar course of the phrenic nerve due to the dense adhesions, but as these lesions were visible on the parietal and visceral pleural surfaces as well as the diaphragm, we believed they would also be in the anterior hilum and most likely responsible for the diaphragmatic paralysis. We reasoned that taking these anterior adhesions down would not have assisted the operation, and so felt that conversion to open was not necessary. The multiple small diaphragmatic fenestrations were mature and fibrotic, preventing repair so this was not performed.

Our patient had a quick recovery with minimal postoperative stay allowing a hospital episode cost advantage. She had significant relief of symptoms and was further managed by the endometriosis clinic to prevent the recurrence of TES. She was treated with a GNRH analogue pre and postoperatively and has been under follow-up as per BSGE guidelines for follow-up of complex endometriosis cases. However, because of poor compliance with HRT due to drug symptoms and her wish to become pregnant, her pleural effusions have recurred requiring regular drainages. She has required further assessment with imaging and a definitive decortication has recently been performed taking down all the adhesions and enabling the lung to expand and fill the space.

We believe that the advantages of RATS described here along with the potential continual upgrade of the robotic system confer the superiority of this technique over VATS for both patient and surgeon. This case showcases RATS as a promising, feasible approach in the surgical treatment of catamenial haemothorax and TES.

## Figures and Tables

**Table 1:** American Reproductive Society of Medicine staging for endometriosis.

Endometriosis stage	Manifestation of the condition
Stage I (1–5 points)	Minimal
	Few superficial implants
Stage II (6–15 points)	Mild
	More and deeper implants
Stage III (16–40 points)	Moderate
	Many deep implants
	Small cysts on one or both ovaries
	Presence of filmy adhesions
Stage IV (>40 points)	Severe
	Many deep implants
	Large cysts on one or both ovaries
	Many dense adhesions

## Data Availability

The data supporting this case report are from previously reported studies, which have been cited within this article.
